# The apple REFPOP—a reference population for genomics-assisted breeding in apple

**DOI:** 10.1038/s41438-020-00408-8

**Published:** 2020-11-01

**Authors:** Michaela Jung, Morgane Roth, Maria José Aranzana, Annemarie Auwerkerken, Marco Bink, Caroline Denancé, Christian Dujak, Charles-Eric Durel, Carolina Font i Forcada, Celia M. Cantin, Walter Guerra, Nicholas P. Howard, Beat Keller, Mariusz Lewandowski, Matthew Ordidge, Marijn Rymenants, Nadia Sanin, Bruno Studer, Edward Zurawicz, François Laurens, Andrea Patocchi, Hélène Muranty

**Affiliations:** 1 Molecular Plant Breeding, Institute of Agricultural Sciences, ETH Zurich, 8092 Zurich, Switzerland; 2 Breeding Research group, Agroscope, 8820 Wädenswil Switzerland; 3 IRTA (Institut de Recerca i Tecnologia Agroalimentàries), 08140 Caldes de Montbui, Barcelona Spain; 4 Centre for Research in Agricultural Genomics (CRAG) CSIC-IRTA-UAB-UB, Campus UAB, 08193 Bellaterra, Barcelona Spain; 5 Better3fruit N.V., 3202 Rillaar, Belgium; 6 Biometris, Wageningen University and Research, 6708 PB Wageningen, The Netherlands; 7 Hendrix Genetics Research, Technology and Services B.V., PO Box 114, 5830AC Boxmeer, The Netherlands; 8 IRHS, Université d’Angers, INRAE, Institut Agro, SFR 4207 QuaSaV, 49071 Beaucouzé, France; 9 ARAID (Fundación Aragonesa para la Investigación y el Desarrollo), 50018 Zaragoza, Spain; 10 Research Centre Laimburg, 39040 Auer, Italy; 11 Department of Horticultural Science, University of Minnesota, St. Paul, MN 55108 USA; 12 Institute of Biology and Environmental Sciences, University of Oldenburg, 26129 Oldenburg, Germany; 13 Research Institute of Horticulture, 96-100 Skierniewice, Poland; 14 School of Agriculture, Policy and Development, University of Reading, Whiteknights, RG6 6AR Reading UK; 15 Laboratory for Plant Genetics and Crop Improvement, KU Leuven B-3001 Leuven, Belgium; 16 Present Address: GAFL, INRAE, 84140 Montfavet, France

**Keywords:** Plant breeding, Plant breeding, Genome-wide association studies

## Abstract

Breeding of apple is a long-term and costly process due to the time and space requirements for screening selection candidates. Genomics-assisted breeding utilizes genomic and phenotypic information to increase the selection efficiency in breeding programs, and measurements of phenotypes in different environments can facilitate the application of the approach under various climatic conditions. Here we present an apple reference population: the apple REFPOP, a large collection formed of 534 genotypes planted in six European countries, as a unique tool to accelerate apple breeding. The population consisted of 269 accessions and 265 progeny from 27 parental combinations, representing the diversity in cultivated apple and current European breeding material, respectively. A high-density genome-wide dataset of 303,239 SNPs was produced as a combined output of two SNP arrays of different densities using marker imputation with an imputation accuracy of 0.95. Based on the genotypic data, linkage disequilibrium was low and population structure was weak. Two well-studied phenological traits of horticultural importance were measured. We found marker–trait associations in several previously identified genomic regions and maximum predictive abilities of 0.57 and 0.75 for floral emergence and harvest date, respectively. With decreasing SNP density, the detection of significant marker–trait associations varied depending on trait architecture. Regardless of the trait, 10,000 SNPs sufficed to maximize genomic prediction ability. We confirm the suitability of the apple REFPOP design for genomics-assisted breeding, especially for breeding programs using related germplasm, and emphasize the advantages of a coordinated and multinational effort for customizing apple breeding methods in the genomics era.

## Introduction

Apple (*Malus* × *domestica* Borkh.) is one of the most economically valuable fruit crops in temperate regions^1^. Thousands of cultivars are grown in national and private repositories around the globe. Extensive genetic variation described in the European apple germplasm illustrates the available genetic diversity among cultivars^2,3^. Only a fraction of the existing apple cultivars is commercially used. Although ongoing breeding programs worldwide aim to create new cultivars adapted to consumer demands and changing climate, these goals could be difficult to reach within the narrow elite genetic pool of modern breeding material^4^.

Since the advent of genomics, genotyping tools have begun to produce affordable genome-wide marker data. Large datasets are being analyzed to explore genotype–phenotype relationships in genome-wide association studies (GWAS) and to allow genomic prediction. Particularly, genomic prediction^5^ has revolutionized breeding and more than doubled genetic progress of major livestock such as cattle^6^. The method relies upon models fitted to broad datasets of genotypes and phenotypes from a training population. The aim is to predict the agronomic performance of breeding material related to the training set based on the marker information alone.

For the application of genomic prediction in fruit trees, apple became a model species due to its economic importance and the range of available research resources^7^. Genomic prediction in apple was tested for the first time by Kumar et al. in 2012^8^. However, this and further studies have been based on a limited number of genetic markers and/or been carried out at a local scale^9–14^.

The prediction accuracy of genomic prediction models generally increases with the number of markers used to genotype the training population, reaching a plateau depending on the architecture of the trait, the number of individuals in the training population, the size of the genome and linkage disequilibrium^15^. Low population structure^2^ and rapid linkage disequilibrium decay in highly diverse apple germplasm^3^ underlie the need for dense SNP marker datasets for GWAS. Considerable progress has been made in the development of genomic resources in apple^16^. Creation of low and medium density SNP arrays, such as the Illumina Infinium^®^ 20K SNP genotyping array (20K array)^17^ was followed by the establishment of the Affymetrix Axiom^®^ Apple 480K SNP genotyping array (480K array) with more than 480K SNPs^18^. Using this SNP array for apple, markers significantly associated with phenological traits in several germplasm collections have been successfully discovered by GWAS^3^. Although the high marker density of the 480K array may lead to higher prediction accuracies than achieved before, no genomic prediction study was conducted using this array so far. Commercial apple breeding programs still cannot afford large-scale genotyping of their germplasm with expensive tools. Therefore, a balance between genotyping density, costs, and predictive ability should be found for the application of genomic prediction in breeding.

Here, we present an apple reference population: the apple REFPOP. The population has been replicated across six environments in Europe and designed for comparing two management practices, which will allow for a thorough and unique study of the effects of genotype, environment, and management as well as their interactions on apple phenotypes. The main objectives of this study were to consolidate the high-density SNP marker dataset for all apple REFPOP genotypes, apply the SNP marker dataset when describing population characteristics of the apple REFPOP and prove suitability of the apple REPOP design for genomics-assisted breeding. Success of the genomics-assisted breeding may depend on characteristics such as marker density, trait architecture or size of the training population^10,15^. For the first time in apple, these aspects could be tested with (i) GWAS and genomic prediction using the high-density 480K array marker dataset, (ii) a comparison of the effects of SNP density on GWAS and genomic prediction, and (iii) the prediction precision analysis. Our further aim was to discuss the use of the apple REFPOP for genomic prediction in multiple environments, across multiple traits and multi-management practices and to facilitate the improvement of apple breeding using the established apple REFPOP.

## Results

### Composition of the apple REFPOP

The apple REFPOP was ultimately built with (i) 269 diploid accessions representing a wide range of genetic diversity in apple, originating from various geographic regions around the globe and (ii) 265 diploid progeny from 27 parental combinations from several European breeding programs (Supplementary Tables 1 and 2). Similar to a previous study^2^, 194 European accessions were classified according to their origin into (i) Northern and Eastern Europe incl. Russia and Baltic countries (NEE, number of accessions *n* = 28), (ii) Western and Central Europe (WCE, *n* = 134), (iii) Southern Europe with accessions from Spain, Italy, and Portugal (SE, *n* = 22), and (iv) Southeastern Europe containing accessions from Romania, Northern Macedonia, Moldova, Bulgaria, and Turkey (SEE, *n* = 10). The 69 non-European accessions originated from (i) Australia and New Zealand (ANZ, *n* = 8), (ii) Canada (CAN, *n* = 16), (iii) Japan (JPN, *n* = 9), (iv) United States of America (USA, *n* = 34), and (v) South Africa (ZAF, *n* = 2). Six accessions previously analyzed with the 480K array but of unknown origin were also included in the apple REFPOP.

### Marker imputation and validation of the imputation output

Missing marker values in the reference set obtained from the 480K array (see “Material and methods” section, Table 1) were inferred through a minor imputation step to obtain a full dataset of 303,239 SNPs covering the 17 apple chromosomes. This minor imputation step was performed for a collection of 1356 accessions (including 269 apple REFPOP accessions) and six progeny genotyped in separate studies^18,19^. Subsequently, a major marker imputation applied to the 259 of the 265 progeny that were genotyped with the 20K array (the remaining 6 progeny with 480K array data available were included in the reference set) increased the marker density from 7060 to 303,239 SNPs (97.7% of marker values imputed). Additionally, imputation accuracy of 0.96 and 0.94 when calculating Pearson correlations between imputed and original values across individuals and across markers, respectively, was estimated for 86 genotypes from two parental combinations, ‘Fuji’ × ‘Pinova’ and ‘Golden Delicious’ × ‘Renetta Grigia di Torriana’.

**Table 1:** Overview of the unique genotypes used in the genotype imputation

Number of unique genotypes	Set of data	Population type	SNP array resolution
269	Reference	Accessions, apple REFPOP	480K
1,087	Reference	Accessions, additional material	480K
6^a^	Reference	1 parental combination, apple REFPOP	480K
259	Imputation	27 parental combinations, apple REFPOP	20K
86^b^	Validation	2 parental combinations, additional material	480K

^a^6 progeny of ‘Fuji’ × ‘Pinova’

^b^40 progeny of ‘Fuji’ × ‘Pinova’ and 46 progeny of ‘Golden Delicious’ × ‘Renetta Grigia di Torriana’

### Linkage disequilibrium and population structure

**Fig. 1:**
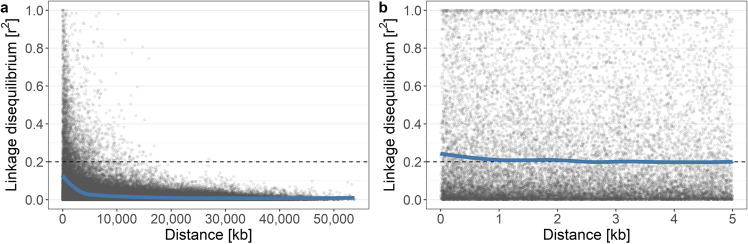
Linkage disequilibrium decay in the apple reference population. Linkage disequilibrium with a loess smoother for **a** distances betweenSNPs across the span of chromosomes, and **b** for SNPs within a 5 kb distance

From the complete set of genome-wide SNP data for both apple REFPOP accessions and progeny, rapid decay of linkage disequilibrium was found (Fig. 1a). The loess smoother fitted to *r*^2^ values of SNPs within a 5 kb distance dropped below a threshold of 0.2 at distance of 2.52 kb, the curve being very flat (Fig. 1b). Average *r*^2^ calculated at 100, 5, 1 kb and 100 bp was 0.14, 0.21, 0.21, and 0.24, respectively. Separate analysis of the accession and progeny group showed a similar pattern of linkage disequilibrium in both groups (Supplementary Figs. 1 and 2).

**Fig. 2:**
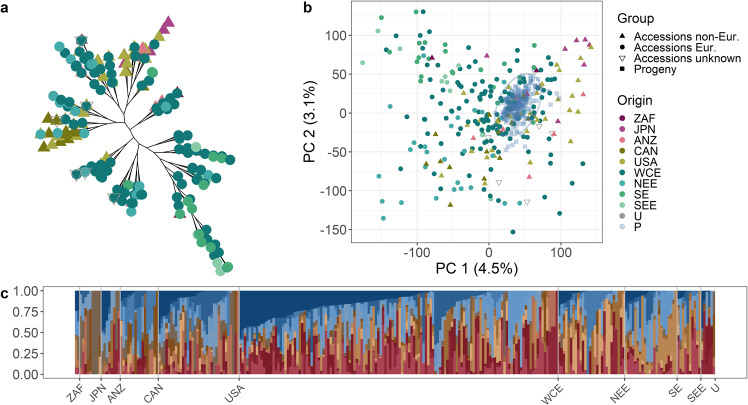
Structure of the apple reference population. **a** Unrooted neighbor-joining tree of the accession group, colors correspond to the legend in “**b**”. **b** Principal component analysis of the accession group with progeny group as supplementary individuals encircled with a normal confidence ellipse (constructed using a multivariate normal distribution, level 0.95). Plot of the first two principal components with their respective proportion of the total variance shown within brackets. **c** ADMIXTURE bar plot of the accession group for *K* = 14. Labels in plots “**a**” to “**c**” refer to the geographic origin of genotypes: ZAF (South Africa), JPN (Japan), ANZ (Australia and New Zealand), CAN (Canada), USA (United States of America), WCE (Western and Central Europe), NEE (Northern and Eastern Europe), SE (Southern Europe), SEE (Southeastern Europe), U (accessions of unknown geographic origin), and P representing the progeny group in plot “**b**”. In plot “**c**”, each group of genotypes with a common geographic origin is labeled at its right side

In an unrooted neighbor-joining tree of apple REFPOP accessions (Fig. 2a), the non-European accessions clustered at the upper side of the tree with a transition towards the European accessions clustering at the lower branches. In the principal component analysis (PCA) of the accession group (Fig. 2b), the first two principal components explained only 7.6% of the total variance in genetic markers. The first principal component showed a slight differentiation between European and non-European accessions with the majority of the non-European accessions positioned on the right side of the plot. The second principal component displayed a weak latitudinal cline in the European accessions with the southern and northern European accessions placed towards the opposite extremes of the second component. Progeny, added to the PCA as supplementary individuals after PCA loadings were estimated, were grouped tightly together among the accessions although they did not form any separate cluster (Fig. 2b). The ADMIXTURE analysis revealed that two local minima of the cross-validation error were reached at the number of clusters *K* = 14 and *K* = 17 (Supplementary Fig. 1). For the first minimum (*K* = 14), genotypes sorted by cluster membership within groups defined by geographic region of origin appeared highly admixed (Fig. 2c). A PCA of the progeny group with 13 parents of the crosses included as supplementary individuals showed that the first two principal components described 10.4% of the total variance in the genomic data and the parents fell among the many small clusters formed by members of distinct parental combinations (Supplementary Fig. 4). From all population structure analyses, we concluded that the apple REFPOP was composed of diverse germplasm with very weak population structure together with high levels of admixture.

### Phenotypic analyses

**Fig. 3:**
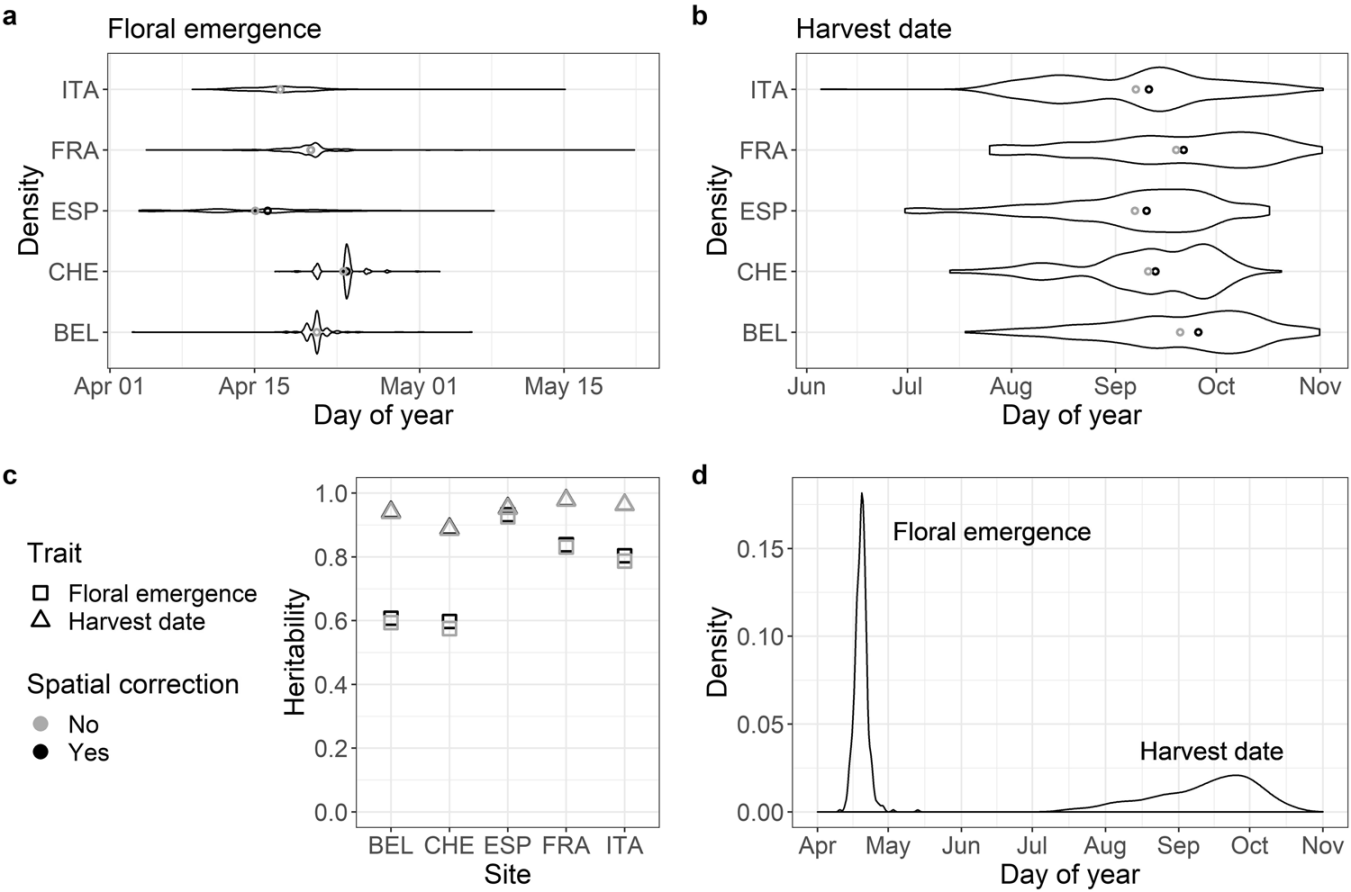
Distributions and heritability of the phenotypic data. **a**, **b** Violin plots of floral emergence and harvest date for individual environments using the adjusted phenotypic values of each tree. Gray and black circles denote mean and median values, respectively. **c** Individual-location clonal mean heritability for two analyzed traits with values before and after the correction of spatial heterogeneity. **d** Density plot of phenotypic least-square means of genotypes across environments with environmental effects removed, calculated from the adjusted phenotypic values of each tree corrected for spatial heterogeneity within environments. The environments were labeled with codes: Belgium (BEL), Switzerland (CHE), Spain (ESP), France (FRA), and Italy (ITA)

Using the spatial analysis of field trials with splines, spatial patterns were captured appropriately for all environments and traits as suggested by the homogeneous residuals (Eq. (1)). Estimated effective dimensions of the spatial model, which are helpful for characterizing the importance of model components^20^, showed a generally larger field variation due to spatial effects for floral emergence than for harvest date (Supplementary Table 3). Differences between environments for both floral emergence and harvest date were visualized using adjusted phenotypic values of each tree in every environment (Fig. 3a, b). Based on this adjusted phenotypic data from 2018, trees began flowering during a narrow period ranging between 16 days in Switzerland and 47 days in France. On average, the flowering began earlier at the southern sites (Spain and Italy). By contrast, harvest dates were distributed across a much longer time span of 99–150 days in Switzerland and Spain, respectively. As with the floral emergence, the mean harvest dates tended to be the earliest in the southern European sites. When comparing the adjusted data to the raw data, we found a small systematic increase in individual-location clonal mean heritability for all environments and both traits (Fig. 3c). Values of the individual-location clonal mean heritability after the adjustment were generally high for both traits and larger for harvest date than for floral emergence. Floral emergence data from Poland with individual-location clonal mean heritability below 0.1 were excluded. Using the adjusted phenotypic values of each tree from the remaining environments to estimate the phenotypic least-square means of genotypes across environments, the obtained values were distributed across a narrow period of 33 days for floral emergence whereas the values of harvest date were distributed over 97 days (Fig. 3d).

**Fig. 4:**
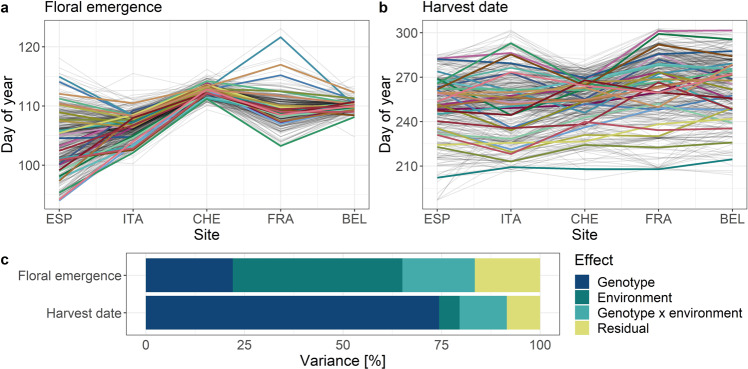
Visualization of variability between traits (floral emergence and harvest date), genotypes (both apple REFPOP groups) and environments. **a**, **b** Predicted values of genotypes adjusted for spatial heterogeneity within each environment, 30 randomly chosen genotypes were highlighted with colors. Order of the environments corresponds to their latitude. The environments were labeled with codes: Spain (ESP), Italy (ITA), Switzerland (CHE), France (FRA), and Belgium (BEL). **c** Stacked bar plots with the variance of the fixed effect of environment and the random effects of genotype, genotype by environment interaction, and residuals; calculated from the model following Eq. (5) (see “Material and methods” section)

Predicted values of genotypes adjusted for spatial heterogeneity within each environment showed that individual genotypes appeared to respond differently to the various environments, with an apparent broader variation among genotypes for harvest date than for floral emergence (Fig. 4a, b). When modeling the effects of environment, genotype and their interaction (see “Material and methods” section, Eq. (5)), the proportion of variance in floral emergence explained by the environment, genotype, and genotype by environment interaction was 43%, 22%, and 18%, respectively (Fig. 4c). For harvest date, the environment explained only 5%, whilst genotype explained 74% and genotype by environment interaction explained 12% of the overall variance (Fig. 4c). Confidence intervals for the estimated variances of random effects of genotype, genotype by environment interaction, and residuals were distinct from zero and therefore, the genotypes differed in the evaluated trait and interacted with the environment (Supplementary Figs. 5 and 6).

### Genome-wide association and prediction analyses

**Fig. 5:**
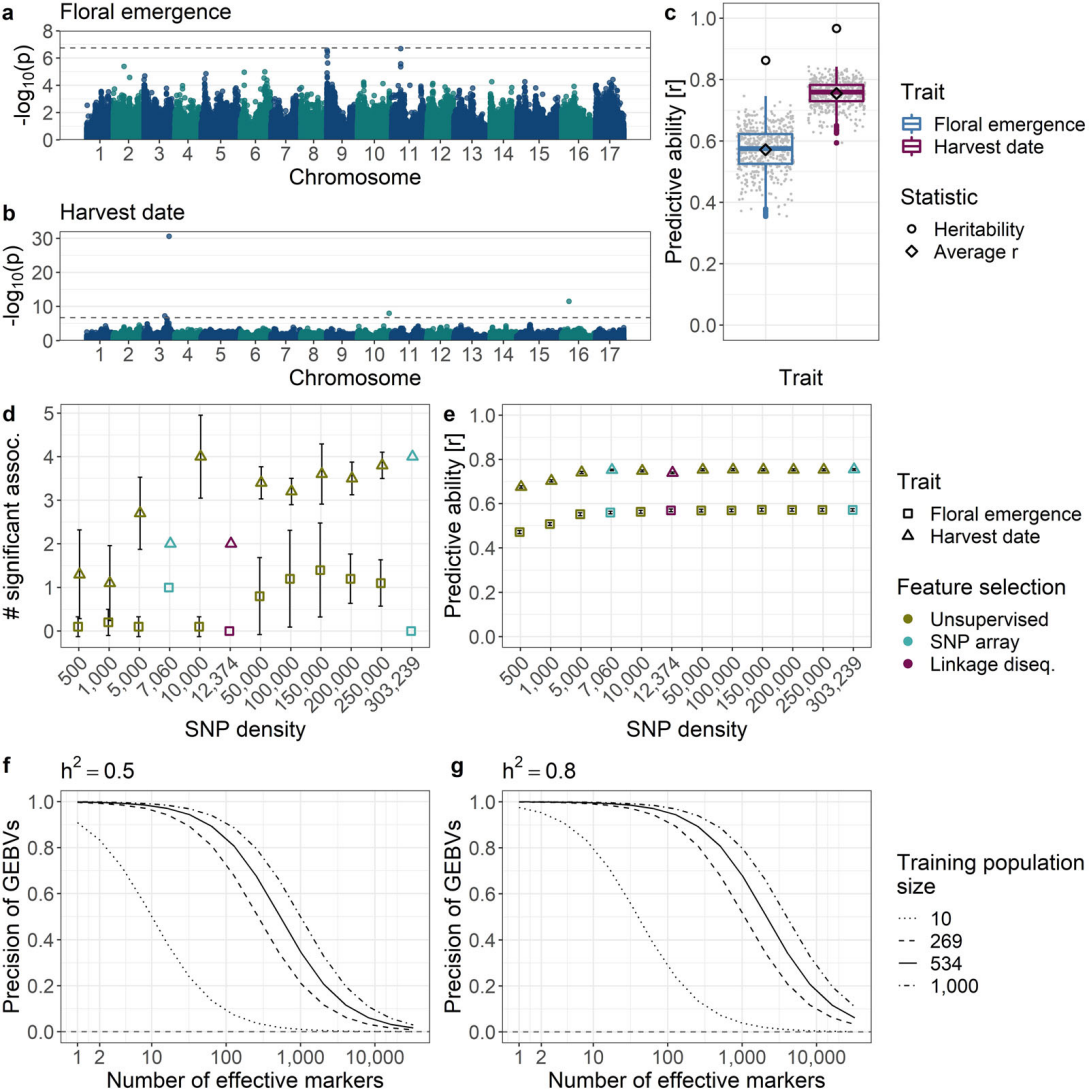
Results of the genome-wide association study (GWAS) and genomic prediction analysis. Manhattan plots for **a** floral emergence and **b** harvest date with log-transformed *p*-values obtained with GWAS and Bonferroni-corrected significance threshold indicated with dashed line. **c** Genomic predictive ability measured with Pearson correlation coefficient, multi-location clonal mean heritability, and average predictive ability. Comparison of the number of significant associations in **d** GWAS and **e** genomic predictive ability measured with the respective mean values and their 95% confidence intervals under various SNP densities obtained through three feature selection strategies (see “Materials and methods” section). Plots “**d**” and “**e**” share a common legend placed in “**e**”. **f**, **g** Expected precision of genomic estimated breeding values (GEBVs) with different training population sizes *N*, number of effective markers *M* in linkage disequilibrium with the genes underlying the trait and two heritability values *h*^2^ of **f** 0.5 and **g** 0.8. The *N* values correspond to a minimum of 10 individuals, and to the sizes of the accession group (*N* = 269), the whole apple REFPOP (*N* = 534) and a population of an approximately double size of the apple REFPOP (*N* = 1000)

The application of GWAS to apple REFPOP dataset identified three markers associated with floral emergence (Fig. 5a, see Supplementary Table 4 for a list of *p*-values). Reported *p*-values were closely below the log-transformed Bonferroni-corrected significance threshold for GWAS performed with the full set of 480K array SNPs; all three associations were significant at density of 150,000 SNPs. Two SNPs were located at the top of chromosome 9 (proportion of explained phenotypic variance *r*^2^ of 0.07 and 0.03) and a third one on chromosome 11 (*r*^2^ = 0.10). Four SNPs were identified to be significantly associated with harvest date using the full set of 480K array SNPs (Fig. 5b, see Supplementary Table 5 for a list of *p*-values). The strongest association was found on the chromosome 3 (*r*^2^ = 0.39) with another significantly associated marker at a distance of 5.3 Mb (*r*^2^ ≈ 0). Two SNPs on chromosome 10 (*r*^2^ = 0.15) and on chromosome 16 (*r*^2^ = 0.11) were also significantly associated with harvest date. The QQ-plots for both traits indicated a good model fit (Supplementary Figs. 7 and 8).

With the full set of 480K array SNPs, floral emergence was predicted using the RR-BLUP model with an average predictive ability of 0.57, whereas the multi-location clonal mean heritability was equal to 0.86 for this trait (Fig. 5c). Average predictive ability of harvest date was 0.75 with the multi-location clonal mean heritability of 0.97 for the trait.

Comparison of GWAS performance under various SNP densities showed that the number of significant associations was higher for harvest date than for floral emergence at all densities (Fig. 5d). For floral emergence, the number of significant associations increased up to density of 150,000 SNPs. Beyond this density, the number of significant associations decreased. The number remained at zero for the 12K marker set (12,374 SNPs) thinned for linkage disequilibrium. For harvest date, the number of significant associations was low (~1) for SNP densities of 0.5–1K. Across all densities larger than 1K, the number of significant associations with harvest date remained high (≥2). For the 12K marker set thinned for linkage disequilibrium, two significant associations on one chromosome were found. Similar to GWAS, predictive ability of genomic prediction was higher for harvest date than for floral emergence at all densities (Fig. 5e). For both traits, the plateau in predictive ability was reached at a density of 10K SNPs and the difference between predictive ability at 500 and 10,000 SNPs was ~0.1.

The precision analysis of genomic prediction (Fig. 5f, g) allowed the assessment of the trait architecture and heritability ranges that may lead to satisfying precision of genomic estimated breeding values. For a training population with a size of *N* = 10, very high precision values (precision ≥ 0.8) may be expected only for simple, oligogenic traits (number of markers underlying the trait *M* ≤ 10). With a training population of the size of the apple REFPOP accession group (*N* = 269), more complex traits with *M* = 100 may be predicted with very high precision if the heritability was high (*h*^2^ = 0.8), but with a decrease in precision for a moderate heritability (*h*^2^ = 0.5). A training population size equal to that of the whole apple REFPOP (*N* = 534) may be sufficient to predict traits with *M* = 100 for both high and moderate heritabilities with very high precision. The precision analysis indicated that highly precise predictions for traits with very complex architecture (100 < *M* ≤ 1000) may be possible if heritability was high and the training population had approximately twice the size of the apple REFPOP (*N* = 1000). However, the increase in estimated precision from *N* = 534 to *N* = 1000 was comparably smaller than the increase from *N* = 269 to *N* = 534.

## Discussion

### Composition of the apple REFPOP

This study is the first report on the apple REFPOP, a reference population created to advance apple breeding with genomics-assisted methods. The population is divided into an accession and a progeny group. Genotypes of its accession group (all diploid) were chosen to represent a wide range of geographic origins and capitalize on the previously available high-density SNP array data. The progeny group stemmed from eight different European breeding programs to represent current tendencies in European apple breeding. For the progeny group, the choice of genotypes was mainly based on genetic distance sampling within progeny groups derived from a number of parental combinations. On the contrary, the choice of genotypes for the accession group did not correspond to a core collection design which generally aims at maximizing genetic diversity^21^. The incorporation of both cultivated accessions and modern breeding material is expected to promote the applicability of the apple REFPOP for genomics-assisted breeding beyond the scope of most current breeding programs, whose genetic bases are generally narrow^22^. Also, since relatedness between training and breeding populations is a crucial factor for successful genomic prediction^13^, the European breeding programs can directly profit from the outcomes of the project because their breeding material has been included in the apple REFPOP. Furthermore, due to the high admixture of the accessions, which stem from across the globe, the levels of diversity in the apple REFPOP appear to be adequate to predict the performance of a broad spectrum of novel breeding material.

### Expanding genomic information via imputation

Lately, resources for high-density marker genotyping in apple became available with the development of the 480K array^18^, but using this array remained costly. Here, we combined the high-density marker information of the accessions with the recently inferred pedigrees for numerous cultivars^19^ to perform a large-scale marker imputation of the progeny group genotyped with the 20K array^17^. Although marker imputation has been routinely applied at large scales in well-studied organisms such as cattle before^23^, here we report for the first time a large scale, high-density imputation in apple. The imputation has multiplied the amount of marker information by ~43× for the progeny group at relatively low cost. A high imputation accuracy was achieved, which is comparable to a similar study in poplar^24^. To evaluate the imputation accuracy, two parental combinations ‘Fuji’ × ‘Pinova’ and ‘Golden Delicious’ × ‘Renetta Grigia di Torriana’ consisting together of 86 progeny with known high-density marker data were used. The parents ‘Fuji’ and ‘Pinova’ are present in the pedigree of five and three parental combinations of the imputation set, respectively. The parent ‘Golden Delicious’ can be found in all of the pedigrees from the imputation set, and therefore, relates to the whole progeny group of the apple REFPOP. Although the pedigree of ‘Renetta Grigia di Torriana’ is unknown, both parental combinations ‘Fuji’ × ‘Pinova’ and ‘Golden Delicious’ × ‘Renetta Grigia di Torriana’ are strongly related to the imputation set and therefore may provide a useful estimate of the imputation accuracy in other imputed parental combinations. An attempt to improve the imputation accuracy might include expanding the reference set with all 19 parents of the parental combinations as well as some of their ancestors for which high-density marker genotype data are not yet available.

### Insights into apple population genomics

Earlier studies with lower density of genome-wide SNPs found that their number of markers was insufficient for GWAS because of a rapid decay in linkage disequilibrium in apple^10,25^. In concordance with previous findings, we found a strong linkage disequilibrium decay over short distances in the apple REFPOP dataset, the pattern of decay being very similar to that described by Urrestarazu et al.^3^ The linkage disequilibrium decay was observed in the apple REFPOP from a distance as short as ~2.5 kb. Given the genome size of a doubled haploid derivative of ‘Golden Delicious’, estimated to 651 Mb^26^, and the number of markers in this study, our marker resolution corresponded to one marker per ~2.1 kb on average. Based on this value, each group of loci in linkage disequilibrium should be on average represented by one marker. Thus, the marker coverage we report seems appropriate for genome-wide analyses, which is supported by our GWAS and prediction analyses. Due to the heterogeneity in the marker density over the genome, the statistical power could be reduced in specific regions with low SNP density.

Partial differentiation between European and non-European accessions in our material is consistent with significant differences between Old World and New World varieties found by Migicovsky et al.^10^ The weak latitudinal cline in the European accessions may reflect the population structure reported in this germplasm by Urrestarazu et al.^2^ The overall weak population structure in the apple REFPOP accession group, which was similar to a previously described weak genetic structure in apples of European origin, presumably reflected the generally highly admixed apple germplasm that is a result of the prominent gene flow characteristic of the cultivated apple gene pool^2,27^.

### An efficient design for genomics-assisted breeding

We applied GWAS using the multi-locus mixed-model (MLMM) method^28^, which accounts for potential confounding effects of kinship and population structure. Thus, this method allows to combine highly related plant material, like in a pedigree-based-analysis, and more diversified, unrelated individuals to integrate different levels of linkage disequilibrium in the same analysis, hence maximizing the chances of finding regions associated with the target trait. By performing GWAS on the apple REFPOP dataset, SNPs associated with phenotypic variation could be identified and assigned to previously known genomic regions. A genomic region located on chromosome 9 was significantly associated with the trait floral emergence. Quantitative trait loci (QTL) at close locations were previously identified for floral bud break, floral emergence, and flowering period^3,29,30^. The second SNP association with floral emergence was located on chromosome 11 approximately 1 Mb downstream from a SNP identified by Urrestarazu et al.^3^ This association may be related to a minor QTL on chromosome 11 discovered using best linear unbiased predictors for genotype by year interaction effect of one season for the mapping of QTLs associated to bud break date (a trait highly correlated to floral emergence) in the study of Allard et al.^30^ For harvest date, one significantly associated SNP on chromosome 3 was located 14,610 bp upstream from the transcription factor NAC18.1 listed as gene MD03G1222600 on the GDDH13 v1.1 genome^26^. Other studies have identified associations between NAC18.1 and harvest date, and the gene is a known member of a family of conserved transcriptional regulators involved in ripening^3,10^. Another SNP on chromosome 3 associated with harvest date was located ~1 Mb upstream from a marker reported before by Urrestarazu et al.^3^ The remaining two SNP associations were found on chromosomes 10 and 16 where QTL for harvest date have also been discovered before^3,31^. The identification of SNPs associated with both phenology traits in well-characterized genomic regions and for one possible minor QTL indicates the suitability of the apple REFPOP for discovering other alleles with large and small effects on trait variability in apple. Although novel marker–trait associations could not be identified and their number was lower than in Urrestarazu et al.^3^, who also used genotypic data of the 480K array, the number of reported marker–trait associations in this work was higher than in GWAS studies using lower SNP densities obtained by genotyping by sequencing^10,32^.

Applying a genomic prediction model with cross-validation, we were able to predict both phenology traits with moderate to high predictive ability when compared to the predictions for different apple traits reported previously^8–11,13^, although cross-validation may have inflated predictive ability compared to a potential independent validation with a test set^33^. To our knowledge, floral emergence has not been predicted in apple before; in this work, an average predictive ability of 0.57 was reached for this trait. The predictions for harvest date had an average predictive ability of 0.75, which was higher than any previously reached accuracies of this, or equivalent, traits in apple^10,11^. The presented genomic prediction methodology may be directly applied for the breeding of floral emergence and harvest date. In particular, breeding programs using germplasm related to the apple REFPOP may capitalize on this work.

The high SNP density in this study allowed for powerful GWAS and genomic prediction analyses, with overall lower performance for floral emergence than harvest date. Floral emergence appeared under weak genetic control with the majority of phenotypic variance explained by the effects of environment and genotype by environment interaction. Markers significantly associated with the trait explained a low proportion of the phenotypic variance in our study, pointing to a complex genetic architecture of floral emergence with many influential genomic regions yet uncovered. With SNP density increasing up to 150,000 SNPs, increasing amount of the phenotypic variance of floral emergence can be explained with GWAS. Environment-specific GWAS or GWAS with phenotypes from across several seasons and locations may allow for improved GWAS performance in floral emergence. For harvest date, a trait under strong genetic control with a large proportion of the phenotypic variance explained by a few major genomic regions, our results suggest that SNP density as low as 10,000 markers may be required to discover genomic associations with the trait. The 10,000 marker subset was also sufficient to reach a plateau of the predictive ability in genomic prediction of both traits, with the difference in predictive ability between traits possibly attributable to the trait architecture. As previously shown in American cranberry^15^, the SNP density at which the plateau of predictive ability is reached may be specific to the apple REFPOP independently of the traits, but may differ for other populations of the same species. These results can have a practical impact on apple breeding, where cost-effective genotyping of 10K SNPs may be sufficient for precise genomic prediction. In breeding research, the same genotyping coverage may be adequate to perform GWAS of oligogenic traits. For complex traits under strong environmental control such as floral emergence, high-density SNP marker datasets remain desirable in GWAS. Future modeling including the effects of environment and genotype by environment interaction may contribute to a higher precision of genomic prediction in complex traits.

In our study, the size of the SNP marker dataset seemed to affect the genomic prediction ability stronger than the feature selection method. On the contrary, a higher power of GWAS to detect marker–trait associations can be obtained with a smaller SNP marker dataset than with a larger set of SNPs depending on the representation of genes underlying the traits in the SNP marker dataset. This way, more marker–trait associations could be revealed with the 7060 SNPs of the 20K array than using SNPs pruned for linkage disequilibrium and such pruning should be avoided prior to GWAS.

In the light of prediction precision analysis, the lower predictive ability for floral emergence may be explained by a higher complexity of this trait, together with lower heritability. Importantly, the results were very likely impacted by the low phenotypic variability of floral emergence in the season of 2018. Additional phenotyping seasons will likely contribute to a better representation of the flowering variability in the population.

### Prospect of the apple REFPOP for multi-environment, multi-management and multi-trait testing

The performance of breeding material in tested as well as untested but similar environments can be predicted accurately using genomic prediction models taking into account genotype by environment interactions^34^. We found a moderate but noticeable effect of the genotype by environment interaction on both phenology traits evaluated in 2018, which contrasts with the limited effect of genotype by environment interaction on a trait similar to harvest date reported in sweet cherry^34^. The replication of the apple REFPOP across six environments will enable the inclusion of these interactions into genomic prediction models. Furthermore, GWAS across separate environments can be performed in the future to identify environment-specific associations and evaluate the stability of associations across environments.

The apple REFPOP was also designed for comparing different management practices: one part of every orchard was grown under the conventional practice of each region, to evaluate the response of the germplasm to environmental effects; the second part can be managed in order to evaluate response to managements such as reduction of irrigation or pesticide application. However, the second management practice has not been applied so far to allow the trees to mature. Incorporating genotype by management and genotype by environment by management interactions in prediction models may help select new material adapted to drier climates or with stronger resistance to apple pests and diseases.

In addition to the multi-environment and multi-management design, protocols for phenotyping of various traits have been applied since 2018 to evaluate the apple REFPOP. More than 10 different traits including yield, fruit quality and phenology are being simultaneously phenotyped with the same method at the six environments. Genotypes are replicated at least twice at each of the environments; each tree is evaluated for all traits separately to allow for variance decomposition up to the individual level (i.e., the tree). Using multi-trait genomic prediction models, prediction of traits with low heritability or labor-intensive phenotyping can be supported by genetically correlated traits with higher heritability and available phenotypes^35,36^.

## Conclusion

This study benefits from a collaborative European approach dedicated to the improvement of apple breeding via genomics-assisted methods. A reference population, which sampled diverse apple germplasm and current European breeding material at larger scales than most current breeding programs, has been established. An extensive set of high-density SNP marker data has been assembled via cost-effective validated marker imputation while making use of the recently available SNP arrays and pedigrees. The imputation method of localized haplotype clustering together with the consolidated high-density SNP marker dataset can be implemented as a standard for cost-effective genomics-assisted breeding. Our diversity and quantitative genetics analyses showed that the reference population is representative of the current apple diversity and breeding material, and that the associated genotypic resources and experimental design allow for the development and application of genomics-assisted breeding methods in apple. This work emphasized the positive effects of high marker density on GWAS and the role of trait architecture in both GWAS and genomic prediction. The apple REFPOP with its unique multi-environment, multi-management and multi-trait design represents a rich source of data for future environment-specific GWAS and genomic predictions. Particularly, the predictions produced with models accounting for the interaction effects between genotype, environment, and management, as well as using multi-trait modeling can help untangle the effects underlying the traits and ultimately improve the efficiency and success of apple breeding. The apple REFPOP will be the cornerstone of many future projects including the application of genomic selection in apple and the work on apple in the EU-H2020-INVITE project (2019–2024).

## Materials and methods

### Composition of apple REFPOP plant material

The apple REFPOP collection composed of accessions and progeny was designed and established by the collaborators of the FruitBreedomics project^7^. The accession group consisted of old and modern diploid accessions representing a wide range of genetic diversity in apple. Simple sequence repeat data obtained by Fernández-Fernández^37^, Lassois et al.^21^ and Urrestarazu et al.^2^ were used to allocate unique genotype code (so-called MUNQ, for *Malus* UNiQue genotype code as described by Muranty et al.^19^) to the accessions included in these studies, which resulted in 1292 unique genotypes available for further choice. When possible, passport data of the accessions belonging to each unique genotype were used to identify its country or region of origin. To form the accession group, a subset of the unique genotypes was created. All possible unique genotypes were chosen when <15 genotypes were available per each country or region of origin. For origins represented by a larger number of accessions, priority was given to the genotypes already analyzed with the Axiom^®^Apple480K array^18^. Additional genotypes of each origin were chosen randomly so that the overall number of selected genotypes per origin was proportional to the number of genotypes of this origin in the whole collection of unique genotypes. Unique genotypes previously analyzed with the 480K array but of unknown origin were also included. Additionally, five accessions (‘Red Winter’, ‘O53T136’, ‘Priscilla-NL’, ‘P7 R4A4’, and X6398) considered as founders in the progeny group pedigree were added to the accession group. Most accessions were chosen from the apple germplasm collection of the National Fruit Collection, Brogdale, United Kingdom in order to simplify collection of the plant material. The budwood of the founders stemmed from two sources: France’s National Research Institute for Agriculture, Food and Environment (INRAE), Angers, France and Wageningen University and Research (WUR), Wageningen, The Netherlands. Availability of budwood from trees affected the numbers of chosen accessions. Therefore, accessions often had to be either (i) replaced with a different accession of the same MUNQ or (ii) excluded in case no other accession corresponding to the unique genotype was available or could provide enough budwood. Additionally, the triploid accession ‘Biesterfelder Renette’ (MUNQ 1106.1) and the accession ‘Karinable’ (MUNQ 7828) with no available SNP data were excluded from the analysis, although planted in the orchards.

The progeny group of the apple REFPOP included 27 full-sib parental combinations previously used in the European project FruitBreedomics^7^. These full-sib parental combinations originated from eight different breeding programs and they were obtained from 32 parents while 13 of the parents were included in the accession group. For most parental combinations, genotypic information was available ahead of this study, with different genotyping density depending on the parental combination. Twenty-two parental combinations had been genotyped with the 20K array^7,17,38^. In addition, in the frame of a pilot study of genomic selection^9^, three other parental combinations were genotyped with a custom 512 SNPs array using the TaqMan OpenArray technology covering the whole genome at a very low density. Finally, for two parental combinations, whole genome data were not available.

A subset of 10 individuals per parental combination was chosen to form the progeny group of the REFPOP. For the 25 parental combinations for whom the whole genome marker data was available, 10 individuals were chosen using a genetic distance sampling strategy^39^. In all cases, the 10 individuals formed the center of each of the clusters defined by genetic distance. Where budwood was not available in sufficient numbers, they were either (i) replaced by individuals closest to the center of the same cluster or (ii) excluded from the progeny group leading to fewer than 10 genotypes per parental combination being chosen (as in the case of parental combinations X6679 × X6808 and X6679 × X6417). From the parental combination ‘Jonathan’ × ‘Prima’, 11 progeny were included in the REFPOP. One progeny of the ‘Dalinette’ × X6681 parental combination (NOVADI/0830) was found as triploid using 20K genotypic data and thus excluded from the analysis, although planted in the orchards. For the two parental combinations without whole genome marker data available, individuals were chosen randomly.

### Multiplication of plant material and planting design

In 2015, budwood from each apple genotype was collected and grafted onto ‘M9’ rootstocks. The grafting was performed in three different nurseries, i.e., at (i) INRAE Angers, France, (ii) Better3Fruit, Rillaar, Belgium, and (iii) Consorzio Italiano Vivaisti, San Giuseppe, Italy. The following year, grafted trees were planted across six contrasting environments, each located in (i) Rillaar, Belgium, (ii) Angers, France, (iii) Laimburg, Italy, (iv) Skierniewice, Poland, (v) Lleida, Spain, and (vi) Wädenswil, Switzerland. The environments ranged across several biogeographical regions in Europe, i.e., the Mediterranean in Spain, Atlantic in France and Belgium, Alpine in Italy and Continental biogeographical region in Switzerland and Poland^40^. Depending on the environment, planting distance within and between tree rows ranged from 0.9 to 1.3 m and 3.2–3.6 m, respectively. All orchards were divided into two parts: the first part was to be managed with the common agricultural practice of the country, the second was to receive alternative management conditions (e.g., low pesticide or water input). So far, both parts have been managed in the same way. The first part consisted of two randomized complete blocks, totaling together 1068 trees, each block containing one replicate per genotype. The second part also consisted of two randomized complete blocks, each containing one representative of approx. one-third of the genotypes (on average 184 genotypes). Every environment shared about half of these genotypes (92 on average) with one of the other countries (country pairs: Belgium–Italy, France–Switzerland, Poland–Spain). Additionally, each environment shared on average 34 genotypes with three further countries. The blocks within parts were used to ensure that two replicates of the same genotype were not planted at a close proximity to each other, but were not intended to block for environmental effects. The two parts were physically separated by a row of trees comprised of additional representatives of genotypes included in the apple REFPOP as well as other material. Since the alternative management regimes were not applied in the initial years, the trees in the barrier, as well as trees in what would later be the alternative management scenario may have contributed phenotypic values for traits in 2018. Three control genotypes ‘Gala’, ‘Golden Delicious’, and Modì^®^ ‘CIVG198’ were each replicated 48 and 18 times in the first and the second part of the orchard, respectively.

### Molecular marker genotyping

A SNP marker dataset for the apple REFPOP was produced using two overlapping SNP arrays of different resolution. The genotypic data generated with the 480K array for a total of 1356 unique genotypes were retrieved from previous studies^3,19^. The SNP marker dataset included all apple REFPOP accessions (including 13 parents of the apple REFPOP progeny group) and additional unique genotypes later used as a reference set for marker imputation. The applied filtering strategy differed from the original one described by Bianco et al.^18^ in discarding the quality prediction based on metrics of the SNP clusters and making use of the pedigree, which was reconstructed in a recent study using all genotyped diploid unique genotypes^19^. For more details about SNP filtering, see Supplementary Methods 1. The SNP positions consistent with the apple reference genome based on the doubled haploid GDDH13 v1.1^26^ were used. Markers unassigned to the 17 apple chromosomes were excluded, resulting in a dataset of 303,239 biallelic SNPs.

Genomic data for the apple REFPOP progeny group were generated using the 20K array. For 210 progeny from 22 parental combinations, the data were already available^7,17,38^. The 49 remaining progeny from parental combinations X338 × ‘Braeburn’, ACW 11303 × ACW 18522, ACW 13652 × ACW 11567, ‘Dalinette’ × X6681, and X6398 × ‘Pinova’ were genotyped with the 20K array within the framework of this study. All 20K array SNPs were filtered and the allele data for the remaining SNPs were curated to ensure the data made logical marker inheritance and co-segregation patterns following the methods and principles described by Vanderzande et al.^41^ The set of 20K-array-generated markers was aligned to the 480K array marker set and 7060 of the 20K array SNPs were retained for further analysis.

### Marker imputation

Due to the difference in marker resolution of the SNP arrays that were used to generate the SNP marker datasets, marker imputation was performed to provide high-density SNP marker information across the whole apple REFPOP. Genotypes included in the apple REFPOP and additional genotypes involved only in the imputation were used either as (i) reference for the imputation or (ii) imputation set or (iii) validation set (Table 1). First, a reference set of 480K array data was formed from 1356 accessions and six progeny of the cross ‘Fuji’ × ‘Pinova’ that had been previously used for validation of the 480K array and the analysis of apple pedigrees^18,19^. Second, the imputation set was formed of the remaining 259 progeny from 27 parental combinations (i.e., including the remaining four progeny from the parental combination ‘Fuji’ × ‘Pinova’ that were not included in the validation set). Third, 40 additional progeny of the parental combination ‘Fuji’ × ‘Pinova’ and 46 progeny of ‘Golden Delicious’ × ‘Renetta Grigia di Torriana’ which had all been genotyped using the 480K array^18^, but none of which had been chosen for inclusion in the apple REFPOP, were designated as a validation set.

The imputation was performed with the localized haplotype clustering implemented in the software Beagle 4.0 using pedigree information^42^. In the first step, reference genotypes (Table 1, reference set of data) along with the recently inferred pedigrees^19^ were supplied to the program for the inference of haplotype phase and minor marker imputation in the reference set. To prepare data for the second step of imputation, SNP density of the validation set (480K array data, see also Table 1, validation set of data) was decreased to the density of the imputation set (20K array data, see also Table 1, imputation set of data) in order to spike the imputation set with known samples of the validation set. In the second step of the imputation, phased reference genotypes along with the pedigrees were used to impute missing marker values in the extended imputation set (i.e., both imputation set and reduced-density validation set samples). Imputation accuracy in the validation set was then evaluated by computing the Pearson correlation between the imputed and original high-density SNP genotypic values in the validation set controls.

### Genomic data analyses

#### Linkage disequilibrium

Linkage disequilibrium statistics were calculated as a square of the correlation coefficient (*r*^2^) between pairs of SNPs on each chromosome with the R package snpStats^43^. To reduce computational time, SNPs of each chromosome were sampled randomly to include one tenth of the markers per chromosome in the *r*^2^ calculation, which resulted in 4.6 × 10^8^ marker combinations. A loess smoother (*α* = 0.5) was fitted to 100,000 randomly chosen *r*^2^ values across the whole span of chromosomes. Additionally, the loess smoother (*α* = 0.5) was calculated for all obtained *r*^2^ values for pairs of SNPs within a 5 kb distance. To determine the distance between SNPs at which the *r*^2^ dropped below 0.2, average *r*^2^ was calculated at 100, 5, 1, and 0.1 kb (100 bp) as a mean of all *r*^2^ within a window of 100 bp around each of the values.

#### Population structure

The neighbor-joining method as implemented in the R package ape^44^ was used to estimate and visualize an unrooted neighbor-joining tree. Principal component analysis (PCA) with supplementary individuals was performed with the R package FactoMineR^45^. Tenfold cross-validation for the number of populations *K* = {1, 2, …, 20} was performed with ADMIXTURE 1.3, a program for estimating ancestry in unrelated individuals^46^. ADMIXTURE was used with default settings and a subset of markers filtered according to the program’s manual. To avoid spurious effects of high linkage disequilibrium between adjacent markers, SNPs were removed in sliding windows of 50 SNPs advanced by 10 SNPs when squared correlation was >0.1 for pairs of variants, leading to a subset of 12,374 SNPs. The correct *K* value was identified at the lowest cross-validation error. As structure is best estimated among unrelated (or weakly related) individuals, the neighbor-joining tree and ADMIXTURE were estimated for the accession group only. For the population structure analyses, the European accessions were divided into several broad regions of origin to compensate for the uncertainty around the exact origin of old varieties^2^.

### Phenotype scoring

Two phenotypic traits were evaluated at the six plantation sites in 2018. Floral emergence of each tree was recorded as the date when 10% of flowers opened^47^. Harvest date was measured as the date when more than 50% of the fruits reached full physiological maturity, as determined by iodine coloration or expert knowledge^47^. Both traits were evaluated for each of the replicate trees individually. Deviation from the phenotyping protocol for harvest date led to exclusion of harvest date recorded in Poland. After measurements, dates were converted to counts of days starting at the beginning of the year in which they were measured.

### Phenotypic data analyses

Raw phenotypic values were corrected for spatial heterogeneity individually within environments to obtain the adjusted phenotypic values of each tree. The corrected as well as uncorrected (raw) phenotypic values were used to estimate the individual-location clonal mean heritability. The adjusted phenotypic values of each tree were further used to fit a mixed model including the effects of genotype, environment and their interaction. The variance of each effect was calculated from the fitted mixed model to estimate multi-location clonal mean heritability and the fraction of phenotypic variation associated with the effects. Finally, the adjusted phenotypic values of each tree were used to obtain phenotypic least-square means of genotypes across environments, i.e., a single mean phenotypic value for each genotype.

#### Correction of spatial heterogeneity

To account for spatial variation of the complete block design, e.g., due to different soil composition or water availability in the orchards, and to predict adjusted phenotypic values of each tree, spatial heterogeneity in the phenotypic data was modeled separately for each environment and trait using the spatial analysis of field trials with splines (SpATS)^20^. To specify the smooth component, a two-dimensional penalized tensor-product of marginal B-spline basis functions based on the P-spline ANOVA approach (PS-ANOVA) was defined with the default settings as a function of covariates plantation row and column, further denoted as *f*(*u, v*). The following linear mixed model was fitted

1 $${\boldsymbol{y}} = f({\boldsymbol{u}},{\boldsymbol{v}}) + {\boldsymbol{Z}}_{\mathrm {g}}{\boldsymbol{c}}_{\mathrm {g}} + {\boldsymbol{Z}}_{\mathrm {r}}{\boldsymbol{c}}_{\mathrm {r}} + {\boldsymbol{Z}}_{\mathrm {c}}{\boldsymbol{c}}_{\mathrm {c}} + {\boldsymbol{\varepsilon }},$$

with ***y*** being the vector of phenotypic values measured for each tree, ***u*** and ***v*** denoting the numeric positions, i.e., rows and columns, the vectors ***c***_g_, ***c***_r_ and ***c***_c_ being the random effect coefficients for the genotypes, rows and columns (as factors) associated with the design matrices ***Z***_g_, ***Z***_r_ and ***Z***_c_, respectively, the ***ε*** denoting the random error vector. Spatial independence assumption of the error vector was visually assessed using a residuals’ spatial plot for each model fit as described by Rodríguez-Álvarez et al.^20^ To characterize the importance of model components, effective dimensions associated with each random factor (***c***_g_, ***c***_r_, and ***c***_c_), the PS-ANOVA spatial trend (*f*_*v*_(***v***)*, f*_*u*_(***u***),***u****h*_*v*_(***v***),***v****h*_*u*_(***u***) and *f*_*u,v*_(***u***,***v***)) and the total effective dimension ED_s_ (sum of partial effective dimensions associated with each component of the PS-ANOVA spatial trend) were assessed^20^. Predicted values of genotypes adjusted for spatial heterogeneity within each environment were produced with random model terms of the smooth component and the random row and column effects excluded from the predictions. These values were used to visualize variability between traits and sites. Residuals for each tree (i.e., each replicate of a genotype) were extracted from the model fit and summed with the corresponding predicted values of genotypes adjusted for spatial heterogeneity within each environment to obtain adjusted phenotypic values of each tree.

#### Broad-sense heritability and phenotypic variation

To evaluate the efficiency of the spatial correction method when comparing raw data with data adjusted for spatial heterogeneity, individual-location clonal mean heritability *H*^2^ was estimated for each of the traits and environments before and after the spatial correction. A random-effects model was fitted for each environment via restricted maximum likelihood (R package lmer^48^)

2 $$y_{ik} = \mu + g_i + \varepsilon _{ik},$$

where *y*_*ik*_ was the *k*th phenotypic value from genotype *i* (adjusted and non-adjusted phenotypic values of each tree), *µ* was the grand mean, *g*_*i*_ was the random effect of the *i*th genotype and *ε*_*ik*_ was the error term. Individual-location clonal mean heritability was calculated from variance components of the model as total genotypic variance $\sigma _{{g}}^2$ over the phenotypic variance $\sigma _{{p}}^2$

3 $$H^2 = \frac{{\sigma _{{g}}^2}}{{\sigma _{{p}}^2}},$$

where the phenotypic variance was calculated from the genotypic variance, error variance $\sigma _\varepsilon ^2$ and the mean number of replications $\bar n_{r}$.

4 $$\sigma _{{p}}^2 = \sigma _{{g}}^2 + \frac{{\sigma _\varepsilon ^2}}{{\bar n_{{r}}}},$$

The individual-location clonal mean heritability was used to eliminate one trial with the heritability value below 0.1.

For the remaining environments, multi-location clonal mean heritability was estimated for each trait with a pooled analysis across environments using mixed-effects models fitted via restricted maximum likelihood (R package lmer^48^)

5 $$y_{ijk} = \mu + g_i + l_j + gl_{ij} + \varepsilon _{ijk},$$

where *y*_*ijk*_ was the *k*th adjusted phenotypic value of each tree from genotype *i* in environment *j*, *µ* was the grand mean, *g*_*i*_ was the random effect of the *i*th genotype, *l*_*j*_ was the fixed effect of the *j*th environment, *gl*_*ij*_ the interaction effect between the *i*th genotype and *j*th environment (random) and *ε*_*ijk*_ was the error term. Then, multi-location clonal mean heritability was calculated using Eq. (3) with the phenotypic variance estimated as

6 $$\sigma _{{p}}^2 = \sigma _{{g}}^2 + \frac{{\sigma _{{{gl}}}^2}}{{n_{\mathrm {l}}}} + \frac{{\sigma _\varepsilon ^2}}{{n_{\mathrm {l}}\bar n_{\mathrm {r}}}},$$

where $\sigma _{{{gl}}}^2$ was the genotype by environment interaction variance and $n_{\mathrm {l}}$ the number of environments.

From the model fit according to Eq. (5), variance of each random effect was calculated. The fraction of phenotypic variation associated with the fixed effect was estimated as the variance of the vector of values predicted from the model fit when all random effects were set to zero. To assess the precision of the random effects, confidence intervals for the variance components were estimated using profiling likelihood method.

#### Phenotypic least-square means

Phenotypic least-square mean of each genotype across environments was estimated from the adjusted phenotypic values of each tree corrected for spatial heterogeneity within the environments. First, a multiple linear regression model was fitted for each trait

7 $$y_{ijk} = \mu + g_i + l_j + \varepsilon _{ijk}$$

where *y*_*ijk*_ was the *k*th adjusted phenotypic value of each tree from genotype *i* in environment *j*, *µ* was the grand mean, *g*_*i*_ was the effect of the *i*th genotype, *l*_*j*_ was the effect of the *j*th environment and *ε*_*ijk*_ was the error term. Second, phenotypic least-square means (or LS-means) of genotypes across environments were calculated with the R package doBy^49^. The phenotypic least square means of genotypes across environments were used later for genome-wide association analyses and genomic prediction.

### Genome-wide association and prediction analysis

#### Genome-wide association study

The multi-locus mixed model (MLMM) method^28^, a stepwise mixed-model regression for mapping complex traits under population structure, was applied to perform GWAS for both apple REFPOP groups together. As a response variable, the phenotypic least-square means of genotypes across environments were used. MLMM was used as implemented in the R package GAPIT 3.0^50^ with default settings and SNPs with low minor allele frequency (0.05) removed. Marker–trait associations were found as significant for *p*-values falling below a Bonferroni-corrected significance threshold α* = *α*/*m* with *α* = 0.05 and *m* representing the number of tested markers. The proportion of phenotypic variance explained by each SNP marker significantly associated to the phenotypic least-square means of genotypes across environments was estimated as a coefficient of determination (*r*^2^). The *r*^2^ was estimated from a simple linear regression model fitted using the numeric marker values of a single SNP as predictor and phenotypic least-square means of genotypes across environments as response.

#### Genomic prediction

The RR-BLUP model^51^ was used for genomic prediction of breeding values. The model was defined as

8 $${\boldsymbol{y}} = {\boldsymbol{WGu}} + {\boldsymbol{\varepsilon }}$$

with the ***y*** being the vector of phenotypic least-square means of genotypes across environments, ***W*** the design matrix relating genotypes to ***y***, ***G*** the SNP matrix, ${\boldsymbol{u}}\sim N(0,{\boldsymbol{I}}\sigma _u^2)$ the vector of SNP marker effects, and ***ε*** the vector of errors. Five-fold cross-validation was applied with the model and data of the whole apple REFPOP, each run masking 20% of the genotypes as validation set. Marker effects (BLUPs) were generated from the remaining 80% of the genotypes to make predictions for the validation set. Predictive ability was estimated as an average value of Pearson correlation coefficient calculated between observed phenotypic least-square means of genotypes across environments from each validation set and predictions for the same genotypes. The cross-validation was repeated 100 times, the five folds being chosen randomly without replacement before each of the repetitions.

#### Comparison of GWAS and genomic prediction performance under various SNP densities

Additionally to the analyses with the full set of 303,239 SNPs produced with the 480K array, different subsets of SNPs were used to perform the GWAS and genomic prediction to investigate the effect of feature selection approaches on model performance. SNPs in subsets were chosen according to three main feature selection strategies, (i) the SNP set from the 20K array (7060 SNPs) available in the full SNP marker dataset, (ii) the SNP set thinned according to linkage disequilibrium (12,374 SNPs, for details see population structure analysis described above), and (iii) an unsupervised SNP choice. For this latter strategy, data subsets were built for densities of 500, 1000, 5000, 10,000, 50,000, 100,000, 150,000, 200,000, and 250,000 markers. As suggested by Bermingham et al.^52^, the markers were chosen evenly spaced from a random starting point. Since the 480K array was designed to cover not only the genic regions, but also to reach a uniform coverage of non-genic regions^18^, the order of the markers on chromosomes was used as a proxy for their physical distance when sampling. For each of the nine SNP densities, the SNP choice was repeated with 10 different seeds resulting in 90 subsets. For these subsets, GWAS and genomic prediction were performed as described above, with the five-fold cross-validation of the genomic prediction being repeated 10 times for each subset. The number of significant associations detected by GWAS was determined as the number of regions containing SNPs with *p*-values falling below a Bonferroni-corrected significance threshold. Mean number of the significant associations in GWAS and mean prediction ability of the genomic prediction as well as their 95% confidence interval were calculated for each number of chosen markers.

#### Analysis of prediction precision

Expectations of the precision of genomic estimated breeding values were approximated based on Eq. (9), i.e., Eq. (5) in the original article by Elsen^53^. The approximation of the precision ($\hat r^2$) of genomic estimated breeding values was determined for different values of parameters influenced by the experimental design (i.e., species, population size and composition, SNP density, environments, or trait architecture) as

9 $$\hat r^2 \cong \frac{{Nh^2}}{{Nh^2 + M(1 - h^2)}}$$

with *M* the number of loci in linkage disequilibrium with genes underlying the trait, *N* the population size, and *h*^2^ the heritability. Parameter values were chosen to encompass and extrapolate beyond the apple REFPOP design: *N* taking values of 10, 269, 534, and 1000, *M* between 1 and 10^6^ and *h*^2^ being equal to 0.5 (moderate) or 0.8 (high). To interpret the output, trait architecture was classified using *M* into oligogenic (*M* ≤ 10), complex (10 < *M* ≤ 100), and very complex (100 < *M* ≤ 1000). Precision of genomic estimated breeding values was considered very high when equal or larger than 0.8. All statistical analyses and data formatting in this article were performed with R^54^ and visualized with the R package ggplot2^55^, unless stated otherwise.

## Supplementary information

Supplementary figures

Supplementary table 1

Supplementary table 2

Supplementary table 3

Supplementary table 4

Supplementary table 5

Supplementary table 6

Supplementary methods

## Data Availability

All SNP genotypic data generated with the 480K array used in this study have been deposited in the INRAe dataset archive (https://data.inrae.fr/) at 10.15454/IOPGYF. All SNP genotypic data generated using the 20K array used in this study have been deposited in the INRAe dataset archive (https://data.inrae.fr/) at 10.15454/1ERHGX. The imputed SNP marker datasets generated during the current study are available from the corresponding author on reasonable request. The phenotypic data generated during the study (phenotypic least-square means of genotypes across environments) are available in the Supplementary Table 6.
