# Supplementary material for: The apple REFPOP—a reference population for genomics-assisted breeding in apple
Source: Hortic Res. 2020 Nov 1;7:189. doi: 10.1038/s41438-020-00408-8 (PMC7603508; doi:10.1038/s41438-020-00408-8)

**The apple REFPOP - a reference population for genomics-assisted breeding in apple**

Michaela Jung, Morgane Roth, Maria José Aranzana, Annemarie Auwerkerken, Marco Bink, Caroline Denancé, Christian Dujak, Charles-Eric Durel, Carolina Font i Forcada, Celia M. Cantin, Walter Guerra, Nicholas P. Howard, Beat Keller, Mariusz Lewandowski, Matthew Ordidge, Marijn Rymenants, Nadia Sanin, Bruno Studer, Edward Zurawicz, François Laurens, Andrea Patocchi, Hélène Muranty

**Supplementary Figures**

**Supplementary Figure 1:** Linkage disequilibrium of the accession section, with a loess smoother for (A) distances between SNPs across the span of chromosomes, (B) for SNPs within a 5 kb distance.


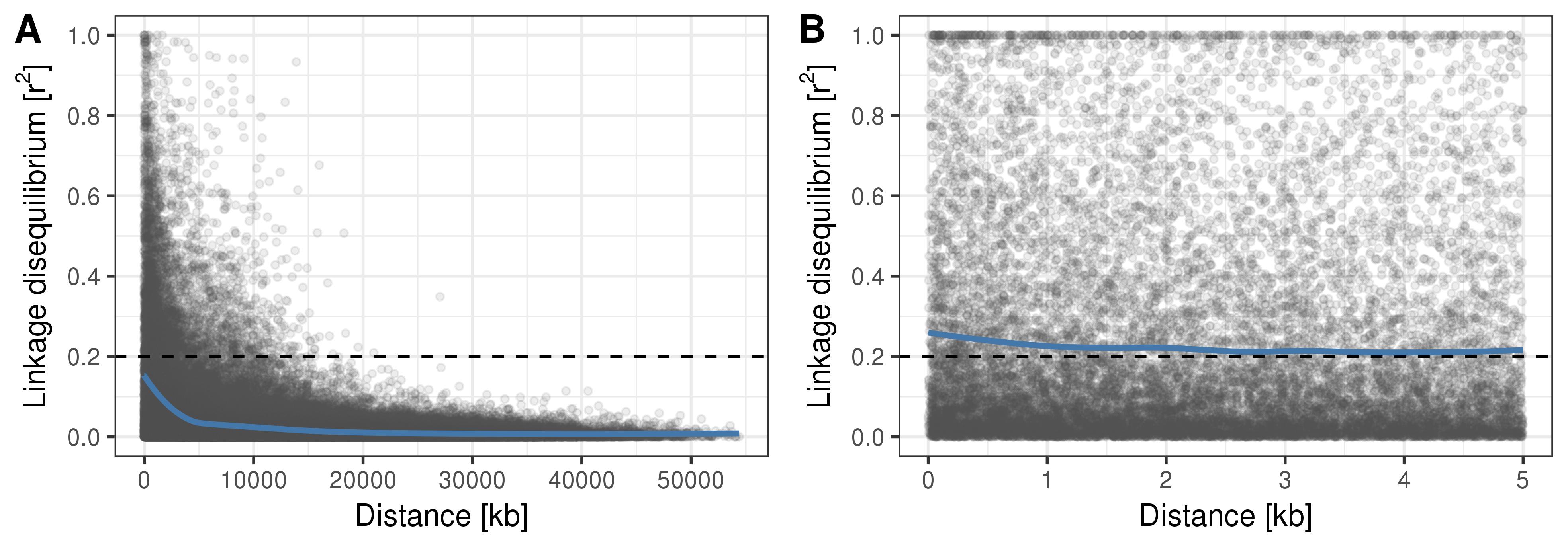


**Supplementary Figure 2:** Linkage disequilibrium of the progeny section, with a loess smoother for (A) distances between SNPs across the span of chromosomes, (B) for SNPs within a 5 kb distance.

**
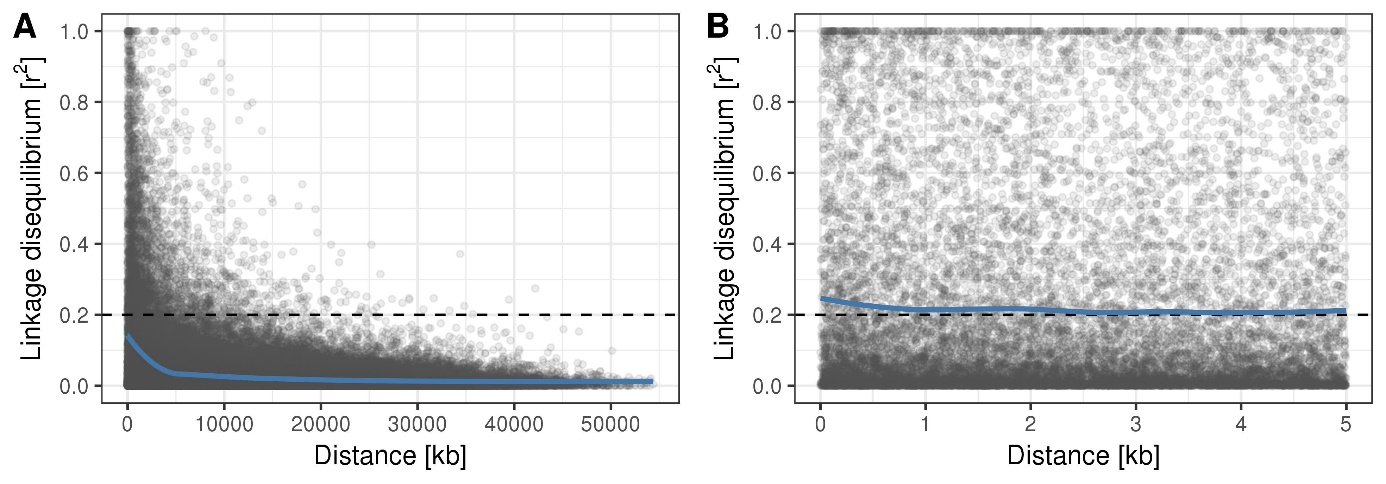
**

**Supplementary Figure 3:** ADMIXTURE cross-validation error


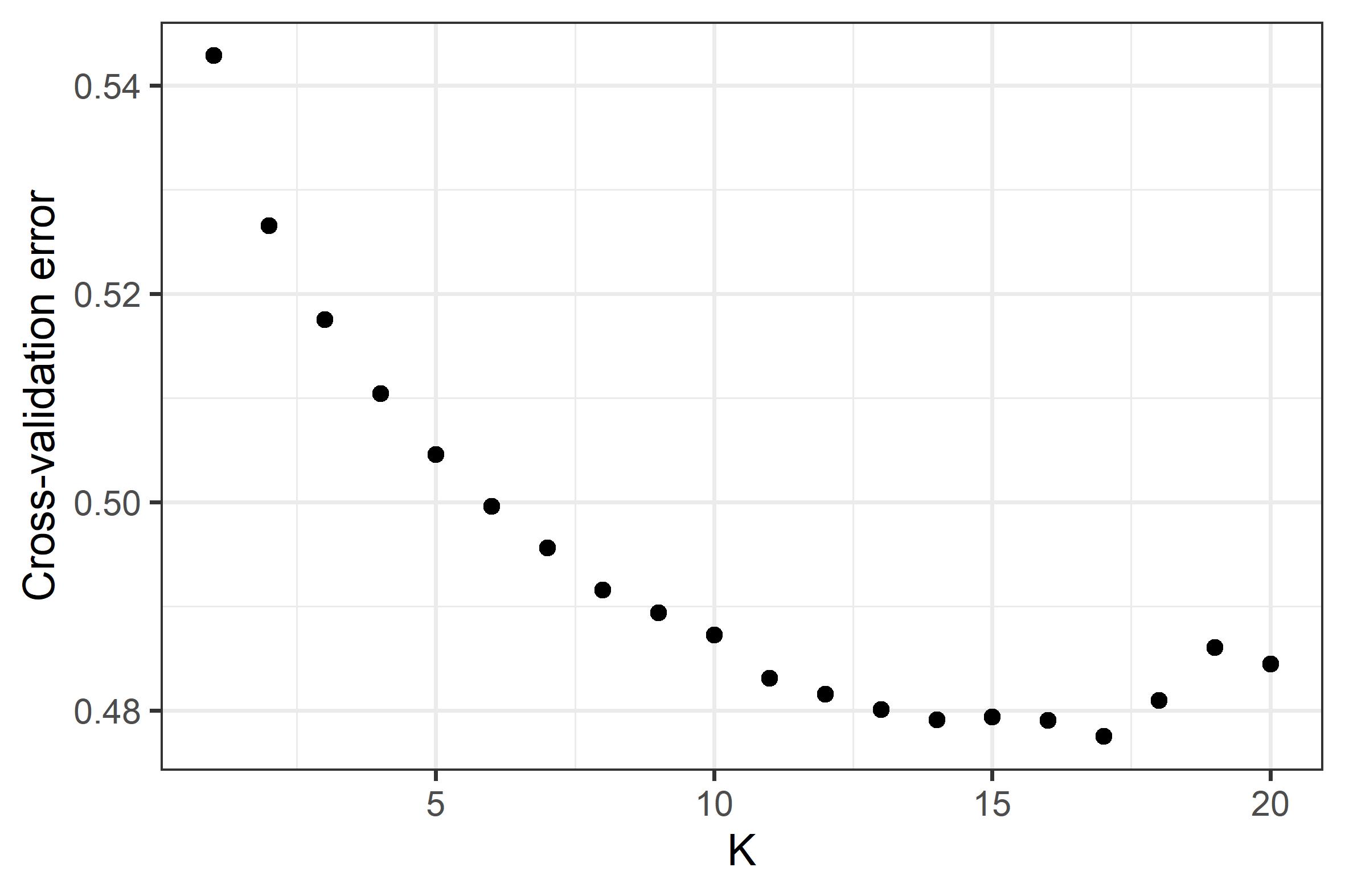


**Supplementary Figure 4:** Principal component analysis of the progeny section (27 parental combinations) with 13 parents as supplementary individuals shown in red color. Plot of the first two principal components with their respective proportion of the total variance shown within brackets.


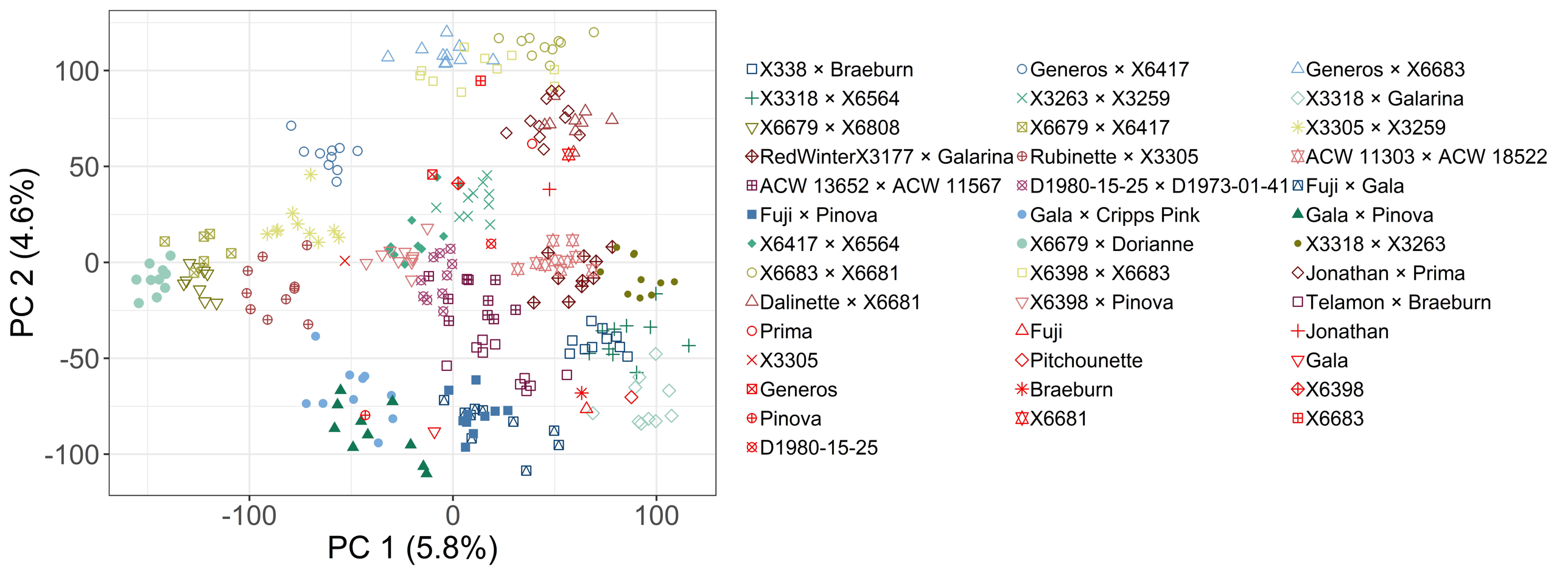


**Supplementary Figure 5:** Standard deviations and confidence intervals for the random effects of genotype (G), genotype by environment interaction (G×E) and residuals (Resid) explaining the floral emergence


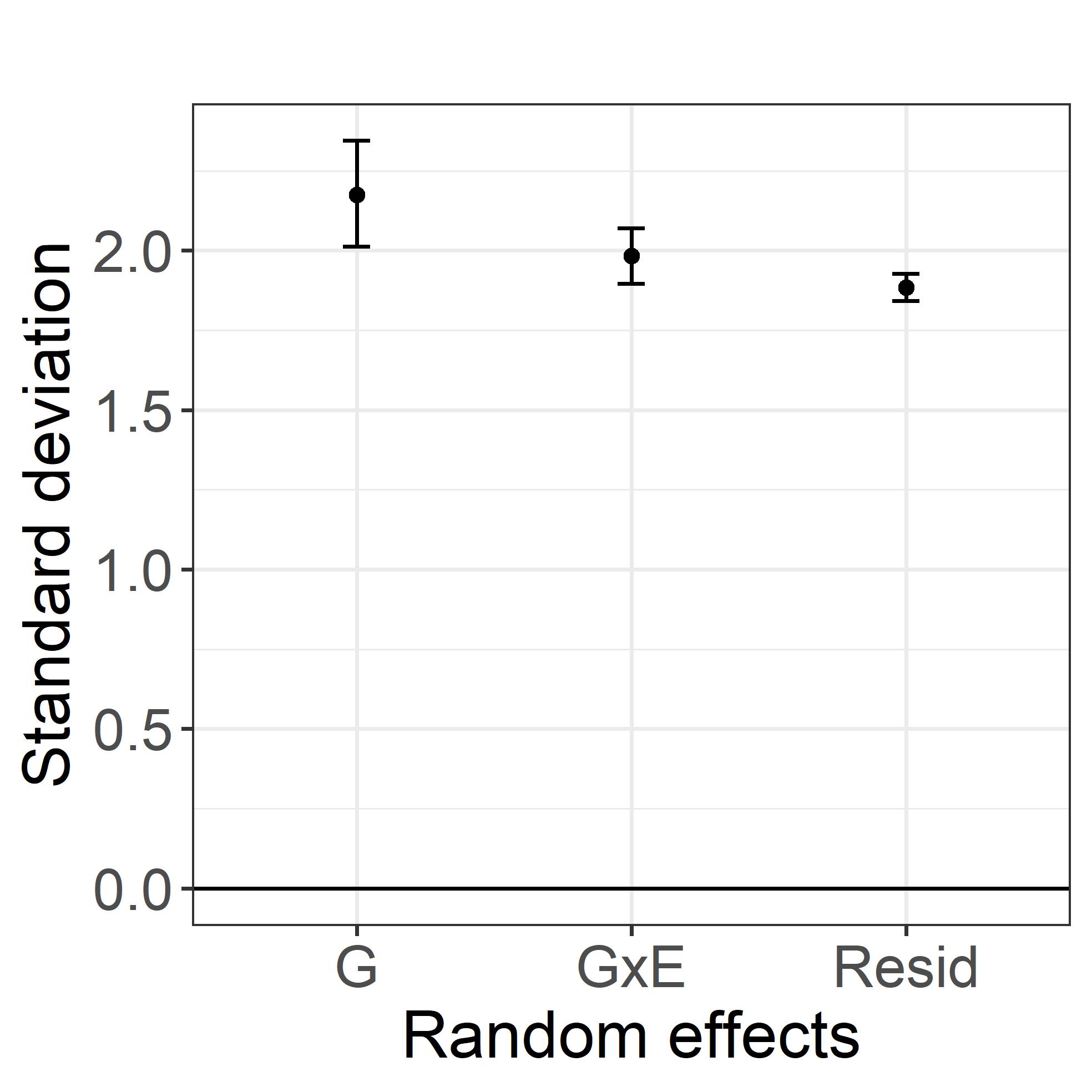


**Supplementary Figure 6:** Standard deviations and confidence intervals for the random effects of genotype (G), genotype by environment interaction (G×E) and residuals (Resid) explaining the harvest date


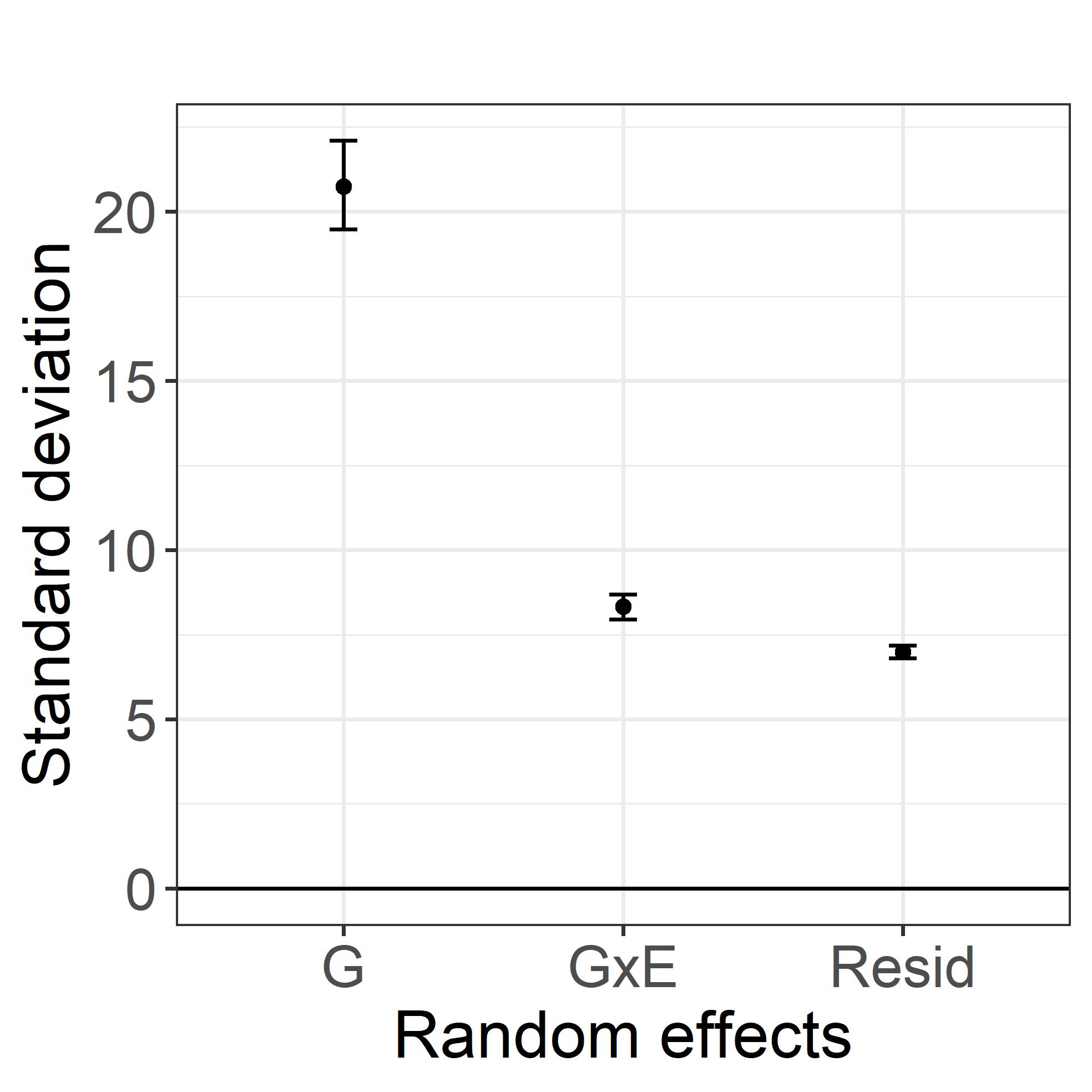


**Supplementary Figure 7:** QQ-plot of the observed versus expected p-values for floral emergence


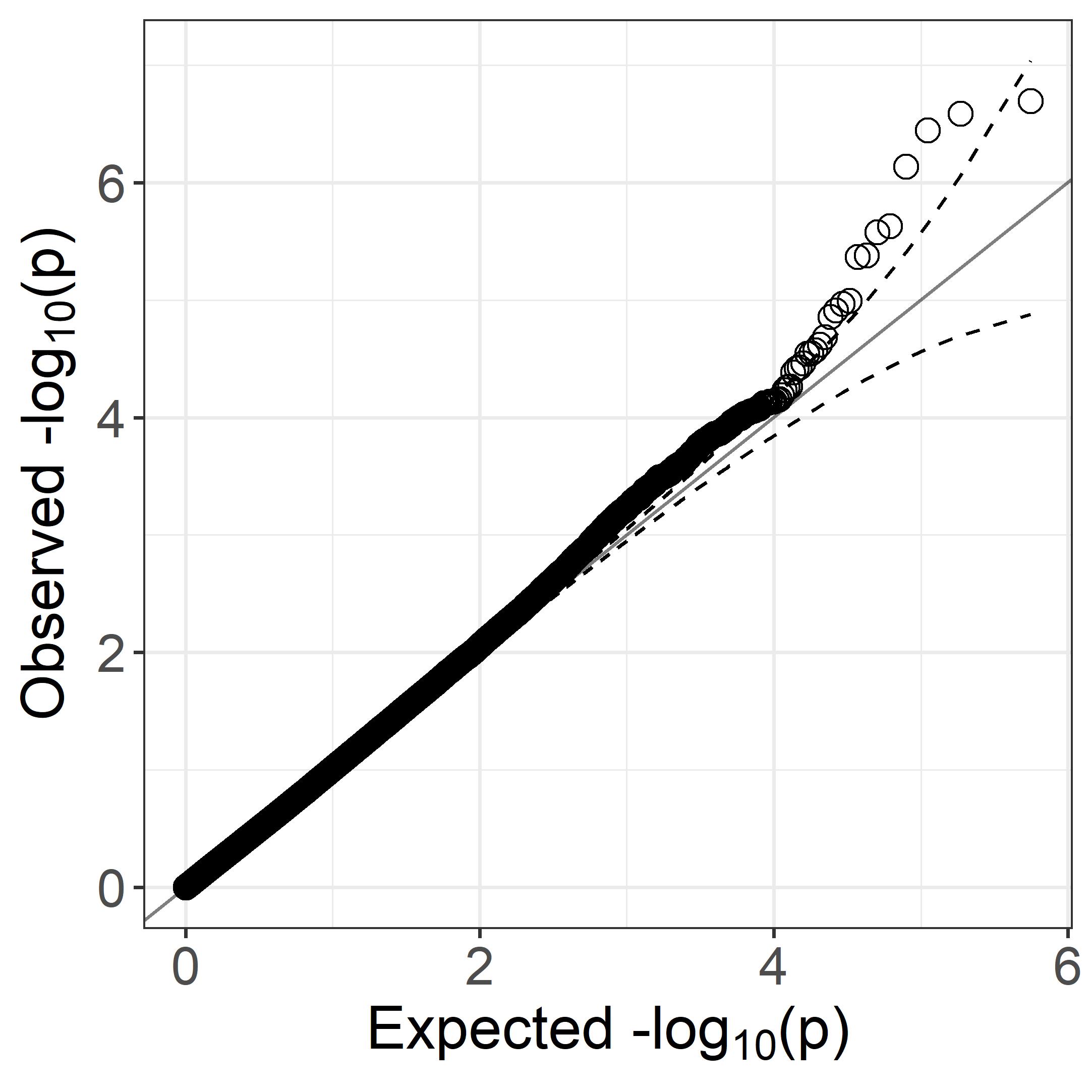


**Supplementary Figure 8:** QQ-plot of the observed versus expected p-values for harvest date


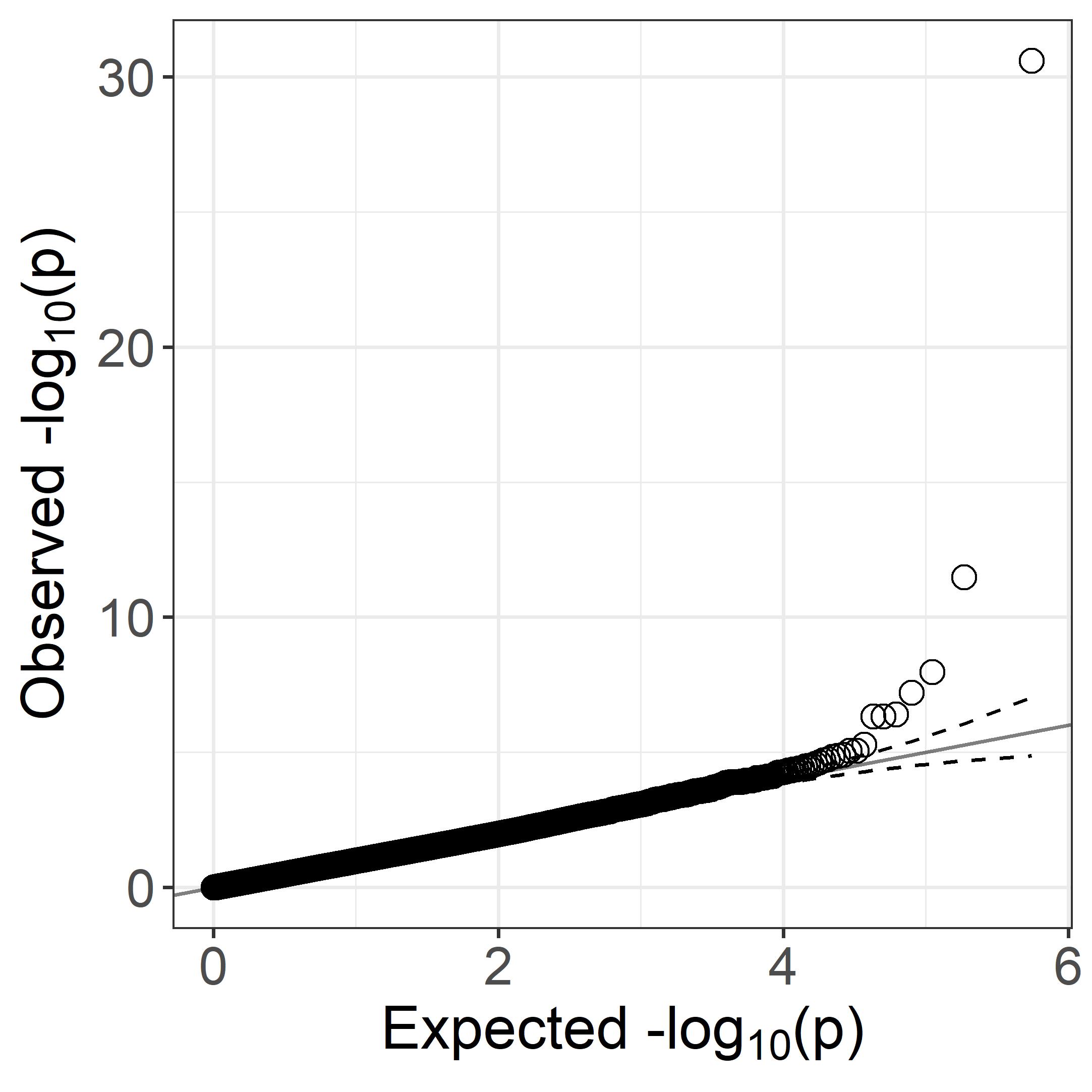

Supplement: Supplementary file 1 — Supplementary figures [file 41438_2020_408_MOESM1_ESM.docx]
