# Supplementary material for: The apple REFPOP—a reference population for genomics-assisted breeding in apple
Source: Hortic Res. 2020 Nov 1;7:189. doi: 10.1038/s41438-020-00408-8 (PMC7603508; doi:10.1038/s41438-020-00408-8)
Supplement: Supplementary file 8 — Supplementary methods [file 41438_2020_408_MOESM8_ESM.pdf]

The apple REFPOP - a reference population for genomics-assisted breeding in apple

Michaela Jung, Morgane Roth, Maria José Aranzana, Annemarie Auwerkerken, Marco Bink, Caroline Denancé, Christian Dujak, Charles-Eric Durel, Carolina Font i Forcada, Celia M. Cantin, Walter Guerra, Nicholas P. Howard, Beat Keller, Mariusz Lewandowski, Matthew Ordidge, Marijn Rymenants, Nadia Sanin, Bruno Studer, Edward Zurawicz, François Laurens, Andrea Patocchi, Hélène Muranty

Supplementary Methods

Supplementary Methods 1

The Axiom Analysis Suite software was used for processing raw hybridization intensity data of all genotyped samples, clustering and genotype calling. Samples with a dish quality control value < 0.82 and sample call rate < 0.97 were excluded. Within the Axiom Analysis Suite software, the SNPs were classified into categories (i) poly high resolution, (ii) mono high resolution, (iii) off target variant, (iv) call rate below threshold, (v) no minor homozygote and (vi) other. The data from the first and fifth category were exported and submitted to further filtering criteria. First, markers were excluded when they showed two or more Mendelian errors in (i) the pedigree identified using all genotyped diploid unique genotypes by Muranty et al. (2020) or (ii) 92 progeny of parental combinations 'Fuji' × 'Pinova' and 'Golden Delicious' × 'Renetta Grigia di Torriana' or (iii) between 'Golden Delicious' and its two doubled haploid offspring. Second, SNPs were removed if they showed (i) differences between duplicates in two or more groups of duplicates (based on 41 groups of duplicates, including 'Golden Delicious' genotyped in each plate), (ii) a heterozygous score in at least one of the two doubled haploid offspring of 'Golden Delicious', (iii) overall heterozygous scoring of five or less.
